# Methylotrophic *Bacillus methanolicus* Encodes Two Chromosomal and One Plasmid Born NAD^+^ Dependent Methanol Dehydrogenase Paralogs with Different Catalytic and Biochemical Properties

**DOI:** 10.1371/journal.pone.0059188

**Published:** 2013-03-19

**Authors:** Anne Krog, Tonje M. B. Heggeset, Jonas E. N. Müller, Christiane E. Kupper, Olha Schneider, Julia A. Vorholt, Trond E. Ellingsen, Trygve Brautaset

**Affiliations:** 1 SINTEF Materials and Chemistry, Department of Biotechnology, Trondheim, Norway; 2 Department of Biotechnology, Norwegian University of Science and Technology, Trondheim, Norway; 3 Institute of Microbiology, ETH Zurich, Zurich, Switzerland; Queen’s University Belfast, United Kingdom

## Abstract

*Bacillus methanolicus* can utilize methanol as the sole carbon source for growth and it encodes an NAD^+^-dependent methanol dehydrogenase (Mdh), catalyzing the oxidation of methanol to formaldehyde. Recently, the genomes of the *B. methanolicus* strains MGA3 (ATCC53907) and PB1 (NCIMB13113) were sequenced and found to harbor three different putative Mdh encoding genes, each belonging to the type III Fe-NAD^+^-dependent alcohol dehydrogenases. In each strain, two of these genes are encoded on the chromosome and one on a plasmid; only one chromosomal *act* gene encoding the previously described activator protein ACT was found. The six Mdhs and the ACT proteins were produced recombinantly in *Escherichia coli*, purified, and characterized. All Mdhs required NAD^+^ as cosubstrate, were catalytically stimulated by ACT, exhibited a broad and different substrate specificity range and displayed both dehydrogenase and reductase activities. All Mdhs catalyzed the oxidation of methanol; however the catalytic activity for methanol was considerably lower than for most other alcohols tested, suggesting that these enzymes represent a novel class of alcohol dehydrogenases. The kinetic constants for the Mdhs were comparable when acting as pure enzymes, but together with ACT the differences were more pronounced. Quantitative PCR experiments revealed major differences with respect to transcriptional regulation of the paralogous genes. Taken together our data indicate that the repertoire of methanol oxidizing enzymes in thermotolerant bacilli is larger than expected with complex mechanisms involved in their regulation.

## Introduction

Methylotrophs use reduced one-carbon (C_1_) compounds such as methane and methanol as their sole sources of carbon and energy [1], [2], [3]. There is both a scientific interest in studying the repertoire of genes and enzymes enabling methylotrophy and applied interest in developing production processes, in which methanol can be converted into useful products, for instance amino acids [4], [5], [6]. Methanol is oxidized by methanol dehydrogenase (Mdh), and an efficient methanol oxidation and concomitant formaldehyde assimilation and dissimilation is of crucial importance for growth and energy generation. In this regard, understanding the nature of the Mdh is important for the basic understanding of methylotrophy and when developing an industrial scale production process using methylotrophic microorganisms.

In methylotrophic bacteria, Mdhs belong to two distinct groups. The best studied group of Mdhs consists of the pyrroloquinoline quinone (PQQ)-containing and cytochrome dependent periplasmic Mdh which exhibits an α_2_β_2_ conformation and is found in Gram-negative bacteria [3], [7]. The other group comprises NAD(P)^+^-dependent cytoplasmic Mdhs composed of one type of subunit that are commonly encoded by Gram-positive methylotrophs, including *Bacillus methanolicus* [7], [8], [9], [10]. In the thermotolerant *B. methanolicus* C1 strain, the *mdh* gene, encoding an NAD^+^-dependent Mdh, was cloned and found to be involved in methanol oxidation [11], [12]. The *B. methanolicus* Mdh primary sequence resembles the iron containing alcohol dehydrogenases, and was therefore classified together with the group III of NAD^+^-dependent alcohol dehydrogenases. Based on electron microscopy, an overall arrangement of a decamer was suggested [11]. Each of the ten subunits was shown to contain a tightly, but non-covalently, bound NAD(H) molecule in addition to a Zn^2+^-ion and 1–2 Mg^2+^-ions [11], [12], [13]. It has been reported that the activity of this Mdh enzyme could be stimulated up to 40-fold by an activator protein, ACT, *in vitro* [13]. ACT belongs to the Nudix hydrolase family [14], [15] and it shows sequence similarity to bacterial ADP-ribose pyrophosphatases (EC.3.6.1.13), enzymes which hydrolyze ADP-ribose, an NADH degradation product [16].

We previously demonstrated that the *mdh* gene in the *B. methanolicus* model strain MGA3 is encoded on a multi-copy natural plasmid, pBM19, and that plasmid-dependent methylotrophy is a widespread trait among *B. methanolicus* strains [4], [17]. The recently sequenced genomes of the strains MGA3 and PB1 [18] surprisingly showed that their chromosomes harbor two additional putative *mdh*-like genes each. In the present report all six Mdh proteins were recombinantly produced in *E. coli*, purified and biochemically characterized. Our results provide new insight into the complex biochemistry and regulation of methanol oxidation in thermotolerant bacilli.

## Materials and Methods

### Biological Materials, DNA Manipulations, and Growth Conditions

The bacterial strains and plasmids used in this study are listed in **Table 1**. *Escherichia coli* DH5α (Invitrogen) was used as the standard cloning host, while proteins were recombinantly expressed in *E. coli* ER2566 (New England Biolabs). *E. coli* strains were generally grown at 37°C in liquid or on solid Lysogeny broth (LB) [19] supplemented with ampicillin (100 µg/ml) when appropriate. Standard recombinant DNA techniques were applied according to Sambrook and Russell [19]. *E. coli* was transformed by the RbCl method (New England Biolabs). Plasmid DNA was isolated by the Wizard Plus SV Minipreps DNA Purification system (Promega), and linear DNA fragments extracted from agarose gel slabs or PCR mixes by the QIAquick Gel Extraction or PCR Purification kits (Qiagen). Genomic DNA was isolated by phenol extraction as previously described [18]. DNA was amplified by the Expand High Fidelity PCR system (Roche Applied Science) and DNA sequencing was performed by Eurofins MWG Operon. *B. methanolicus* cells were grown at 50°C in 100 ml of MeOH_200_ medium containing 200 mM methanol or in Mann_10_ medium containing 20 g/liter d-mannitol [20].

**Table 1:** Bacterial strains and plasmids used in this study.

Strain or plasmid	Description^a^	Reference(s) or source
*B. methanolicus* MGA3	Wild type strain ATCC53907.	[38]
*B. methanolicus* PB1	Wild-type strain NCIMB13113.	[17]
*E. coli* DH5α	General cloning host.	Bethesda Research Laboratories
*E. coli* ER2566	Carries chromosomal gene for T7 RNA polymerase.	New England Biolabs
pGEM-T	*E. coli* cloning vector; Ap^r^, 3001 bp.	Promega
pLITMUS28	*E. coli* cloning vector, Ap^r^, 2823 bp.	Promega
pET21a	*E. coli* expression vector, six-His tag, T7 promoter, Ap^r^, 5443 bp.	Novagen
pTMB1	pLITMUS28 derivative with the MGA3 *mdh2* gene PCR-amplified and cloned intothe NcoI-BamHI sites, Ap^r^, 4005 bp.	This study
pTMB2	pLITMUS28 derivative with the MGA3 *mdh3* gene PCR-amplified and cloned intothe NcoI-BamHI sites, Ap^r^, 4007 bp.	This study
pET21a-mdh (MGA3)	pET21a derivative with the MGA3 *mdh* gene PCR-amplified and cloned intothe NdeI-XhoI sites, Ap^r^, 6513 bp.	This study
pET21a-mdh2 (MGA3)	pET21a derivative with the MGA3 *mdh2* gene PCR-amplified and cloned intothe NdeI-XhoI sites, Ap^r^_,_ 6522 bp.	This study
pET21a-mdh3 (MGA3)	pET21a derivative with the MGA3 *mdh*3 gene PCR-amplified and cloned intothe NdeI-XhoI sites, Ap^r^, 6522 bp.	This study
pET21a-act (MGA3)	pET21a derivative with the MGA3 *act* gene PCR-amplified and cloned intothe NdeI-XhoI sites, Ap^r^, 5922 bp.	This study
pET21a-mdh (PB1)	pET21a derivative with thePB1 *mdh* gene PCR-amplified and cloned intothe NdeI-XhoI sites, Ap^r^, 6516 bp.	This study
pET21a-mdh1 (PB1)	pET21a derivative with the PB1 *mdh1* gene PCR-amplified and cloned intothe NdeI-XhoI sites, Ap^r^, 6516 bp.	This study
pET21a-mdh2 (PB1)	pET21a derivative with the PB1 *mdh*2 gene PCR-amplified and cloned intothe NdeI-XhoI sites, Ap^r^, 6522 bp.	This study
pET21a-act (PB1)	pET21a derivative with the PB1 *act* gene PCR-amplified and cloned intothe NdeI-XhoI sites, Ap^r^, 5922 bp.	This study
pET21a-nudF	pET21a derivative with the *B. subtilis nudF* gene PCR-amplified and cloned intothe NdeI-XhoI sites, Ap^r^, 5922 bp.	This study

a Ap^r^, ampicillin resistance; Cm^r^, chloramphenicol resistance.

### Construction of Expression Vectors

Due to the high sequence similarity between the *mdh2* and *mdh3* genes of MGA3 (here denoted *mdh2* ^M^ *and mdh3* ^M^), they were amplified from MGA3 total DNA by primers binding in the flanking regions of the respective genes. The *mdh2* ^M^ *-*region was amplified using 5′-AACCATGGATGAGGAGGATGTTTGTATGAC-3′ and 5′-TGGATCCTCTTCGTCTTTGGCGAATTAC-3′, while the *mdh3* ^M^ *-*region was amplified using 5′-AACCATGGCAAACAAAGGGGATGTATGTATG-3′ and 5′-AGGATCCCCTCCGTTTTGTCGTATTAC-3′. The DNA fragments were digested by NcoI and BamHI and ligated into the corresponding sites of pLITMUS28 resulting in plasmids pTMB1 and pTMB2, carrying *mdh2* ^M^ and *mdh3* ^M^ respectively. Next *mdh2* ^M^ and *mdh3* ^M^ were PCR amplified from plasmids pTMB1 and pTMB2, respectively while *mdh* ^M^ and *act* ^M^ were PCR amplified from *B. methanolicus* MGA3 total DNA. The *mdh* ^P^, *mdh1* ^P^, *mdh2* ^P^, and *act* ^P^ genes were PCR amplified from PB1 total DNA, and *nudF* was amplified from *B. subtilis* 168 total DNA. The following PCR primer pairs were used: 5′-CATATGACAACAAACTTTTTCATTCC-3′ and 5′-CTCGAGCATAGCGTTTTTGATGATTTGTG-3′ (*mdh* ^M^, 1149 bp); 5′-CATATGACAAACACTCAAAGTGC-3′ and 5′-CTCGAGCATCGCATTTTTAATAATTTGG-3′ (*mdh2* ^M^, 1163 bp); 5′-CATATGAAAAACACTCAAAGTGCATTTTAC-3′ and 5′-CTCGAGCATAGCGTTTTTGATGATTTGTG-3′ (*mdh3* ^M^, 1165 bp); 5′-AAACATATGGGAAAATTATTTGAGG-3′ and 5′-AAACTCGAGTTTATTTTTGAGAGCCTCTTG-3′ (*act* ^M^, 570 bp); 5′-ATACATATGACGCAAAGAAACTTTTTCATTC-3′ and 5′-ATACTCGAGCAGAGCGTTTTTGATGATTTG-3′ (*mdh* ^P^, 1164 bp); 5′-ATACATATGACTAAAACAAAATTTTTCATTC-3′ and 5′-ATACTCGAGCAGAGCGTTTTTGATGATTTG-3′ (*mdh1* ^P^, 1164 bp); 5′-ATACATATGACAAACACTCAAAGTATATTTTAC-3′ and 5′-ATACTCGAGCATAGCATTTTTAATAATTTGTATAAC-3 (*mdh2* ^P^, 1170 bp); 5′-TTTTCATATGGGAAAATTATTTGAGGAAA-3′ and 5′-TTTTCTCGAGTTTATTTTTGAGAGCCTCTTG-3′ (*act* ^P^, 572 bp), 5′-TTTTCATATGAAATCATTAGAAGAAAAAACAATTG-3′ and 5′-TTTTCTCGAGTTTTTGTGCTTGGAGCGCTT-3′ (*nudF*, 572 bp). The ACT and *nudF* PCR products were digested by NdeI and XhoI, and ligated into the corresponding sites of pET21a, in frame with the His_6_-tag sequence, resulting in pET21a_ACT and pET21a_NudF plasmids (see **Table 1**). The Mdh PCR products were directly A/T-ligated into the general cloning vector pGEM-T (Promega). The resulting vectors were completely digested by XhoI and partially by NdeI, and the full-length Mdh-encoding fragments were ligated into the corresponding sites of plasmid pET21a in frame with the His_6_-tag sequence, yielding pET21a_MDH plasmids (**Table1**). All the constructed vectors were verified by sequencing and transferred into the expression host *E. coli* ER2566. The MGA3 and PB1 draft genome sequences are available at Genebank under accession codes ADWW 01000000 and AFEU 01000000 [18].

### Affinity Purification of Recombinant Proteins

The six Mdh proteins, two ACT proteins and NudF were expressed in *E. coli* ER2566 (harboring pET21a_MDH/pET21a_ACT/pET21a_NudF plasmids) and purified essentially as previously described [21]. Protein concentrations were measured spectrophotometrically in a NanoDrop spectrophotometer, (Nano Drop Technologies) using molecular weight and extinction coefficient settings calculated for the His_6_-fusion proteins by the Expasy Prot Param tool (expasy.org/tools/protparam.html) [22] (data not shown). The protein purity was analyzed by sodium dodecyl sulfate-polyacrylamide gel electrophoresis (SDS-PAGE) [19]. Purified proteins were snap frozen in liquid N_2_ and stored at −80°C.

### Enzyme Assays

Alcohol dehydrogenase activities were measured spectrophotometrically in 1 ml cuvettes essentially as previously described [15], [23], and unless otherwise stated, the reaction mixture contained: 100 mM Glycine-KOH pH 9.5, 5 mM MgSO_4_, 0.5 mM NAD^+^, and 0.5 M alcohol (methanol, ethanol, propanol, 1,3-propanediol, or butanol) or alternatively 0.05 M alcohol (pentanol and hexanol), as well as purified Mdh protein (5–100 µg/ml). NAD^+^ was substituted with 0.5 mM NADP^+^, FMN^+^, or FAD^+^ when indicated. When measuring formaldehyde reductase activity, the reaction mixture contained: 50 mM Potassium-phosphate buffer pH 6.7, 0.15 mM NADH, 1 mM DTT and 11.6 mM formaldehyde or acetaldehyde. Unless otherwise stated, the assay components were mixed in the cuvette and pre-warmed to 45°C. The generation or consumption of NADH was monitored at 340 nm. One unit (U) of Mdh activity was defined as the amount of enzyme needed to produce or consume 1 µmol NADH per minute under the conditions described above. Purified ACT (0.1–40 µg) or NudF (20 µg) proteins were added to the reaction mixtures as indicated in the text. The six Mdh proteins were analyzed for pH and temperature optima. All enzymes displayed the highest catalytic activity at pH between 9.5 and 10. The Mdhs were assayed at pH 9.5 at temperatures ranging from 25°C to 45°C, and the results showed that they all had temperature optima around 45°C (data not shown). Because of technical limitations of the instrument used, kinetic experiments were conducted at 45°C.

In order to determine the Michaelis Menten constants of the enzymes during the oxidation of methanol or reduction of formaldehyde, activities were measured at varying methanol concentrations (0.1–2000 mM) keeping NAD^+^ at saturation (0.5 M), at varying NAD^+^ concentrations (1–2000 µM) keeping methanol at saturation (0.5 M), or at varying formaldehyde concentrations (0.1–40 mM) keeping NADH at saturation (0.15 mM). The data were plotted and fitted using Prism 5.04 (GraphPad software). The Mdh reaction has previously been found not to follow Michaelis-Menten kinetics, but rather displays a ping-pong type of reaction mechanism [24]. By using a definition of *K_m_* as the substrate concentration resulting in a reaction rate of ½*V_max_* rough estimates of the *K_m_* values for methanol were made, and they correlated well with the constants found fitting the Michaelis-Menten equation in Prism 5.04. It was therefore decided to report the Prism 5.04 calculated constants.

### Isolation of Total RNA, cDNA Synthesis and Quantitative PCR

Quantitative PCR (qPCR) experiments were performed essentially as described previously [21]. Total RNA was isolated from MGA3 and PB1 cell cultures growing exponentially (OD_600_ = 1.0) with d-mannitol or methanol as the sole carbon source, using the RNAqueous kit (Ambion). Quality control of the RNA, the cDNA synthesis and the qPCR experiments were preformed essentially as described before [18], [21] using the following primers: 5′-ATTCCACCAGCCAGCGTAAT-3′ and 5′-CTTAGCTCCAATTTGCTTAAGTCTTG-3′ (Mdh^M^); 5′-GGATACATGTCAAACACTCAAAGTGC-3′ and 5′-TCTAGACACCATCGCATTTTTAATAATTTGG-3′ (Mdh2^M^); 5′-GGATACATGTAAAACACTCAAAGTGC-3′ and 5′-TCTAGACACCATAGCATTTTTAATAATTTGGATG-3′ (Mdh3^M^); 5′-TCCACCAGCTAGCGTAATTGG-3′ and 5′-AACCTGTGCCATGAAGAAATGC-3′ (Mdh^P^); 5′-TCCATCATCCACTGTATTTGG-3′ and 5′-ACCTGTGCTGTGAAGGAATGC-3′ Mdh1^P^); 5′-CGTGAAGCTGGTGTGGAAGTATT-3′ and 5′-TCCAAACCTTCTGCGACGTT-3′ (Mdh2^P^). Relative quantification of the genes of interest was performed by normalizing the results, relative to 16S rRNA (endogenous control) and a calibrator sample, using a comparative Ct method (2-ΔΔCt method) as previously described [20], [21], [25]. The relative differences in transcript levels of the genes were estimated by calculating the ΔCT values given as follows: ΔCT*_mdh2_* ^M^ = (Ct*_mdh2_* ^M^ – Ct*_mdh_* ^M^), ΔCT*_mdh3_* ^M^ = (Ct*_mdh3_* ^M^ – Ct*_mdh_* ^M^), ΔCT*_mdh1_* ^P^ = (Ct*_mdh1_* ^P^ – Ct*_mdh_* ^P^), and ΔCT*_mdh2_* ^P^ = (Ct*_mdh2_* ^P^ – Ct*_mdh_* ^P^). The primer efficiency of the six genes was tested before the quantitative qPCR experiments were performed.

## Results and Discussion

### Methanol Dehydrogenase Paralogs in the *B. methanolicus* wild-type Strains MGA3 and PB1

**Figure 1:**
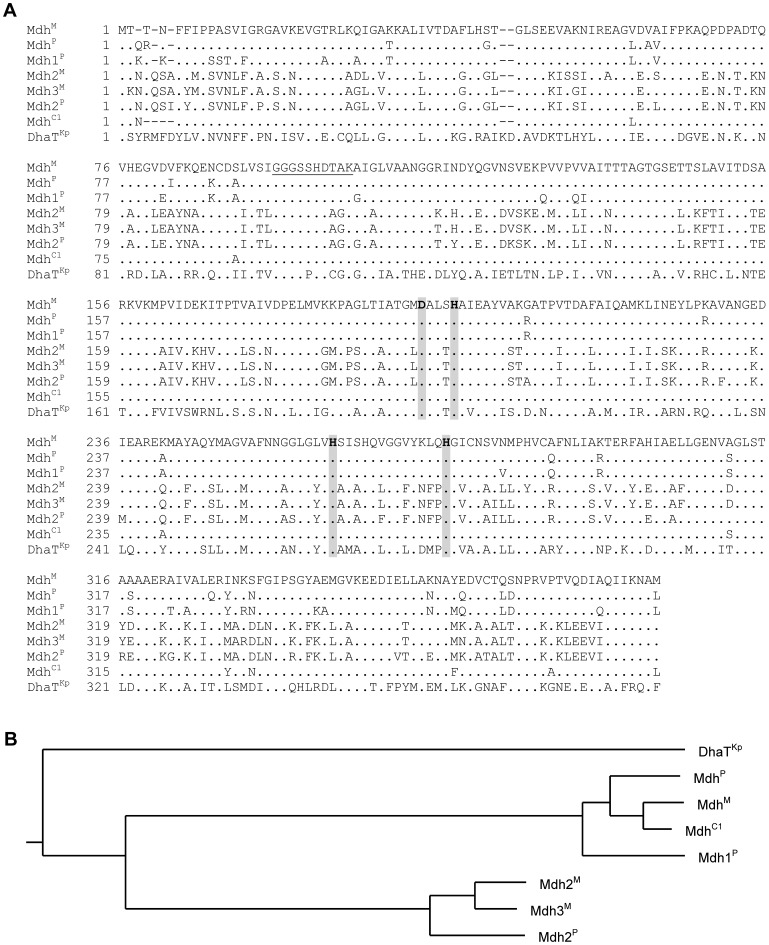
Comparison of the *B. methanolicus* Mdhs and the *K. pneumoniae* 1,3-PDH (DhaT^Kp^). The MGA3 Mdhs are denoted Mdh^M^, Mdh2^M^, and Mdh3^M^; the PB1 Mdhs are denoted Mdh^P^, Mdh1^P^, and Mdh2^P^, while the C1 Mdh is denoted Mdh^C1^. **(**A**)** Primary sequence alignments show a conserved GGGSX_2_DX_2_K motif involved in NAD^+^ binding (underlined) and residues assumed to be involved in metal ion binding (bold and highlighted). Residues in bold are unique for type III Adhs [12]. (B**)** Relationship among the primary protein sequences of the *B. methanolicus* MGA3, PB1 and C1 Mdhs and the *K. pneumoniae* 1,3-PDH, represented by a phylogenetic tree.

The *B. methanolicus* strains MGA3 and PB1 exhibit substantially different macrokinetic properties with respect to methylotrophic growth and were recently both genome sequenced [18]. Notably, both model strains revealed the presence of methanol dehydrogenase homologs. The *B. methanolicus* MGA3 genome sequence [18] showed that in addition to the previously described pBM19-encoded *mdh*-gene (here denoted *mdh* ^M^) [17], two more putative Mdh encoding genes, here denoted *mdh2* ^M^ and *mdh3* ^M^, were present distantly located on the chromosome. Primary sequence alignment of the deduced Mdh2^M^ and Mdh3^M^ proteins revealed that they are 96% identical to each other, and 61% and 62% identical, respectively, to Mdh^M^ (**Figure 1a**). Inspection of the PB1 genome sequence confirmed the presence of three putative MDH encoding genes (here denoted *mdh* ^P^, *mdh1* ^P^ and *mdh2* ^P^). The *mdh* ^P^ gene located on plasmid pBM20 encodes an Mdh^P^ protein sharing 93% primary sequence identity to the Mdh^M^ protein (**Figure 1a**). The *mdh1* ^P^-gene encodes an Mdh1^P^ protein with 92% primary sequence identity to the Mdh^P^ protein, while the *mdh2* ^P^ gene encodes an Mdh2^P^ protein with 91% and 92% primary sequence identity to the Mdh2^M^ and Mdh3^M^ proteins, respectively, while only about 60% primary sequence identity to Mdh^P^, Mdh1^P^ and Mdh^M^. The chromosome of each of the *B. methanolicus* strains contained a single *act*-gene each (here denoted *act* ^M^ and *act* ^P^), encoding the previously described activator protein important for Mdh activity [15], [23]. Based on these sequence analyses, it seems as if MGA3 and PB1 possess two subtypes of Mdh encoding genes; the “*mdh/mdh1”* type and the “*mdh2*/*mdh3* type”. MGA3 has one *mdh*/*mdh1* type gene (pBM19) and two *mdh2*/*mdh3* type genes (chromosome), while PB1 has two *mdh*/*mdh1* type genes (pBM20 and chromosome) and one *mdh2*/*mdh3* type gene (chromosome) (**Figure 1b**). The biological impact of these phylogenetic differences was further investigated and is described below.

### The *B. methanolicus* Mdhs belong to the type III Fe-NAD^+^-dependent Alcohol Dehydrogenase Superfamily

In silico analyses of the deduced Mdh sequences using the Basic Local Alignment Search Tool (BLAST) [26] indicated that they belong to the type III alcohol dehydrogenases (Adhs) [12], which is a super-family of iron-containing Adhs. The best hit with a known 3D structure was the *Klebsiella pneumoniae* DhaT-protein (pdb:3bfj [27]), a 1,3-propanediol dehydrogenase, (1,3-PDH, EC.1.1.1.202), which displayed 45–53% amino acid sequence identity with the Mdhs (**Figure 1a**). Structural models of the MGA3 Mdh^M^ and Mdh2^M^ proteins were made using the fully automated protein structure homology-modeling server SWISS-MODEL (http://swissmodel.expasy.org/) [28], [29], [30]. The gapped BLAST searches [31], [32] resulted in 9 common template hits with E values varying from 1·e^−98^ (pdb:3bfj) to 1·e^−17^ (pdb:1oj7) for Mdh and from 1·e^−112^ (pdb:3bfj) to 2·e^−14^ (pdb:1oj7) for Mdh2. Due to the high homology between the deduced primary sequences of the Mdh^M^, Mdh^P^ and Mdh1^P^ proteins, and between the Mdh2^M^, Mdh3^M^, and Mdh2^P^ proteins, no model search was performed for the remaining Mdhs. Superposing the 9 template files using Deep view/Swiss pdb viewer [33] showed that they all have very similar C_α_-traces, despite the low primary sequence identity (only 26% sequence identity between pdb:3bfj and pdb:1oj7), (data not shown). This implies that the 3D fold of the Mdhs is conserved, and that their varying properties are likely to be caused solely by amino acid variations in and around the catalytic site and/or the NAD^+^ binding site. The 1,3-PDH is a type III Fe-NAD^+^-dependent alcohol dehydrogenase that catalyzes the conversion of 3-hydroxypropionaldehyde into 1,3-propanediol (1,3-PD). It displays a decameric, quaternary structure analogous to the *B. methanolicus* C1 Mdh (here denoted Mdh^C^) [11]. The monomers of the 1,3-PDH folds into two structural domains that are separated by a cleft. The N-terminal domain contains the binding site of the NAD^+^ cofactor and the C-terminal domain includes the residues involved in iron binding [27]. A conserved GGGSX_2_DX_2_K motif involved in NAD^+^ cofactor binding was found in the N-terminal region of Mdh^C^ [23] (in position 95–104 of Mdh^M^, see **Figure 1a**) and this region is also found in the 1,3-PDH and in all Mdhs investigated in this study. The 258–290 region of Mdh^C^ contained several His residues, which were predicted to be involved in metal binding [23]. This is in good agreement with the findings in the *K. pneumoniae* 1,3 PD dehydrogenase, where 4 residues responsible for coordination of the iron metal were found. These residues are conserved in all Mdhs examined in this study and correspond to residues Asp193, His197, His262 and His276 in Mdh^M^ (**Figure 1a**), and are probably responsible for zinc binding in this enzyme.

### The Recombinantly Produced Mdh Proteins from MGA3 and PB1 all Catalyze NAD^+^-dependent Dehydrogenase Reactions with a Wide Range of Alcohols, Including Methanol

The *mdh* ^M^, *mdh2* ^M^, *mdh3* ^M^, *act^M^*, *mdh* ^P^, *mdh1* ^P^, *mdh2* ^P^, act^P^ and *nudF* coding sequences were PCR amplified and cloned into the *E. coli* vector pET21a, in-frame with a 3′-His_6_-tag encoding sequence to simplify purification (**Table 1**). The *nudF* gene encodes a nudix hydrolase in *B. subtilis*, shown to belong to the same protein family as the *B. methanolicus* ACT [15]. All constructed expression vectors were transferred into *E. coli* ER2566, and the resulting recombinant strains were cultivated in shake flasks for production of the respective recombinant proteins. The proteins were purified by affinity chromatography to more than 95% purity as judged from SDS-PAGE (data not shown).

**Figure 2:**
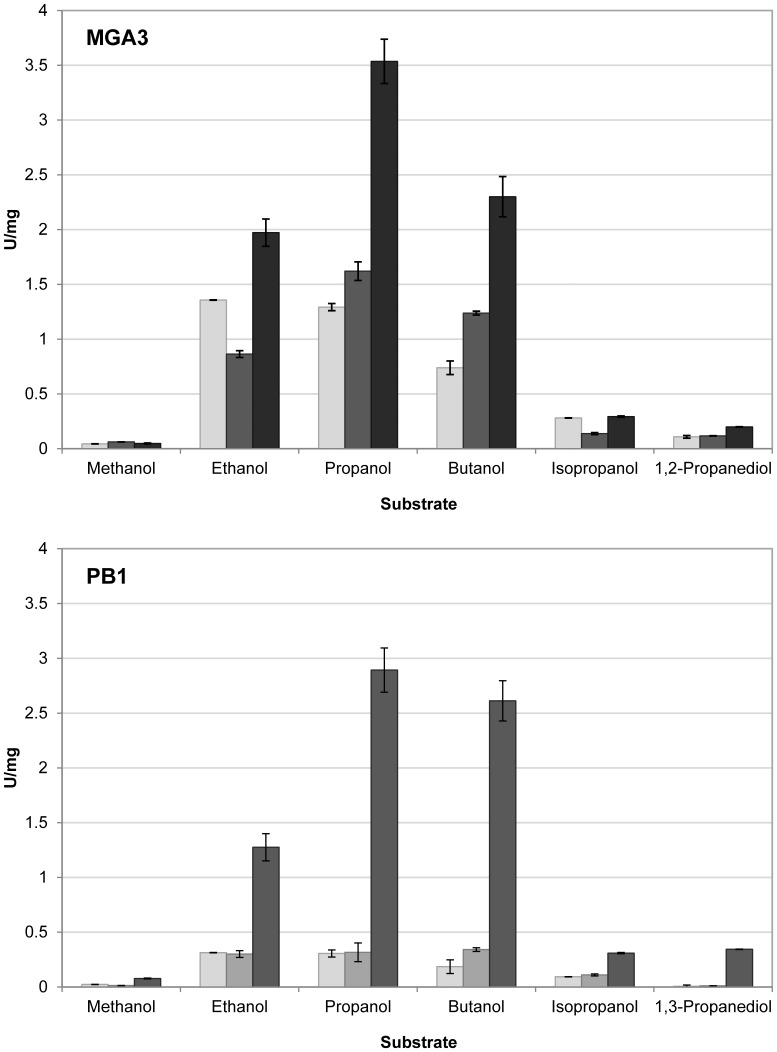
In vitro substrate specificity of *B. methanolicus* Mdhs. MGA3 enzymes are shown in the upper panel, while PB1 enzymes in the lower panel. Catalytic activities of purified Mdh^M^/Mdh^P^ (white), Mdh1^P^ (light grey), Mdh2^M^/Mdh2^P^ (dark grey) and Mdh3^M^ (black) on various alcohols (500 mM) is shown. The data were calculated from the mean value from two independent experiments, performed in triplicate, and the standard errors are included.

The six purified putative Mdh proteins were assayed using different alcohols as substrates and shown to be catalytically active on all alcohols tested (see below), including methanol (**Figure 2**). To rule out if the Mdh proteins can use alternative cofactors, the assay was repeated by substituting NAD^+^ with FAD^+^, FMN^+^ and NADP^+^. In all cases no catalytic activity was detected (data not shown), confirming that none of these alternative cofactors could be used by the Mdhs under the conditions tested. These results demonstrated that the *B. methanolicus* strains MGA3 and PB1 have three different NAD^+^-dependent Mdh-like encoding genes each; one located on a plasmid and two located on the chromosome.

### All Mdh-like Enzymes have Broad Substrate Specificities and Different Preferences for Alcohols

The purified Mdh proteins were tested for catalytic activities using several alternative alcohol substrates, and all enzymes displayed activities on ethanol, propanol, butanol, isopropanol, 1,2-propanediol and 1,3-propanediol (**Figure 2**), when assayed with 500 mM. In addition, due to the low solubility of larger primary alcohols, the enzymes were assayed with 50 mM alcohol concentrations and found to also be active on pentanol and hexanol. The relative catalytic activities for the recently discovered subgroup of Mdh-like enzymes were all shown to be substantially higher with the alcohols tested compared to methanol, similarly to the originally described Mdh of *B. methanolicus* [34]. This may imply that the catalytic site of the Mdhs is easily accessible for larger substrates, and that the substrate binding of medium sized primary alcohols could be more efficient than binding of the smaller methanol. The relative catalytic activities on the different alcohols tested varied substantially between the six Mdhs, indicating different substrate preferences among the proteins. For example, the activities of the Mdh3^M^ and the Mdh2^P^ enzymes with propanol were about 35 to 70-fold higher than their activity on methanol. Notably, the Mdhs belonging to the Mdh2/3 type from both strains displayed higher catalytic activity than the enzymes belonging to the Mdh/Mdh1 type with most of the substrates, under the conditions tested. All six enzymes also displayed formaldehyde and acetaldehyde reductase activities, which is further described below. Based on these data, these proteins could be classified as Adhs rather than Mdhs; however, we prefer to keep the designation of Mdhs for these enzymes, in line with the original Mdh from *B. methanolicus* C1 and MGA3 [15], [17], [23].

### Mdh3^M^ and Mdh2^P^ Display Higher Temperature Stabilities than the Remaining Mdhs

**Figure 3:**
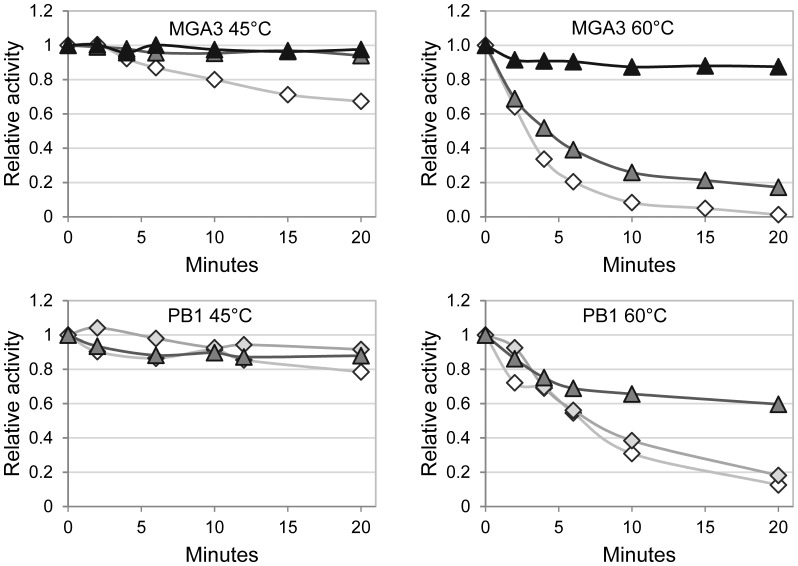
Temperature stability of the MGA3 and PB1 Mdhs. Enzymatic activities of Mdh^M^/Mdh^P^ (white diamond), Mdh1^P^ (light grey diamond), Mdh2^M^/Mdh2^P^ (dark grey triangle) and Mdh3^M^ (black triangle) were measured upon preincubation at 45°C or 60°C. Activity without preincubation was for each enzyme arbitrarily set to 1. The results from at least two independent experiments with standard errors below 10%, and mean values are given.

The heat stability of the six Mdhs was tested by pre-incubation of the proteins at 45°C and 60°C, and samples were taken at different time points for enzyme assays. As expected, all enzymes retained essentially all catalytic activity upon preincubations at 45°C (**Figure 3**). The catalytic activities of Mdh^M^, Mdh2^M^, Mdh^P^ and Mdh1^P^ were however strongly reduced (up to 80%) upon preincubations at 60°C for 6 minutes, while this treatment had only moderately negative effects on the Mdh3^M^ and Mdh2^P^ catalytic activities (**Figure 3**). A selection of the experiments was repeated in the presence of equal amounts of purified ACT; however, ACT addition had no effect on temperature stability for any of the Mdhs (data not shown). One might speculate whether the presumably higher instability of MDH could be an important way of regulating the Mdh levels in the cells to avoid formaldehyde accumulation in response to varying methanol concentrations in the surroundings. However, it could not be ruled out that the stabilities of the Mdhs in vivo are quite different from those measured in vitro.

### The Dehydrogenase, and not the Reductase, Activities of all Six Mdh Proteins are Stimulated by ACT in vitro

**Figure 4:**
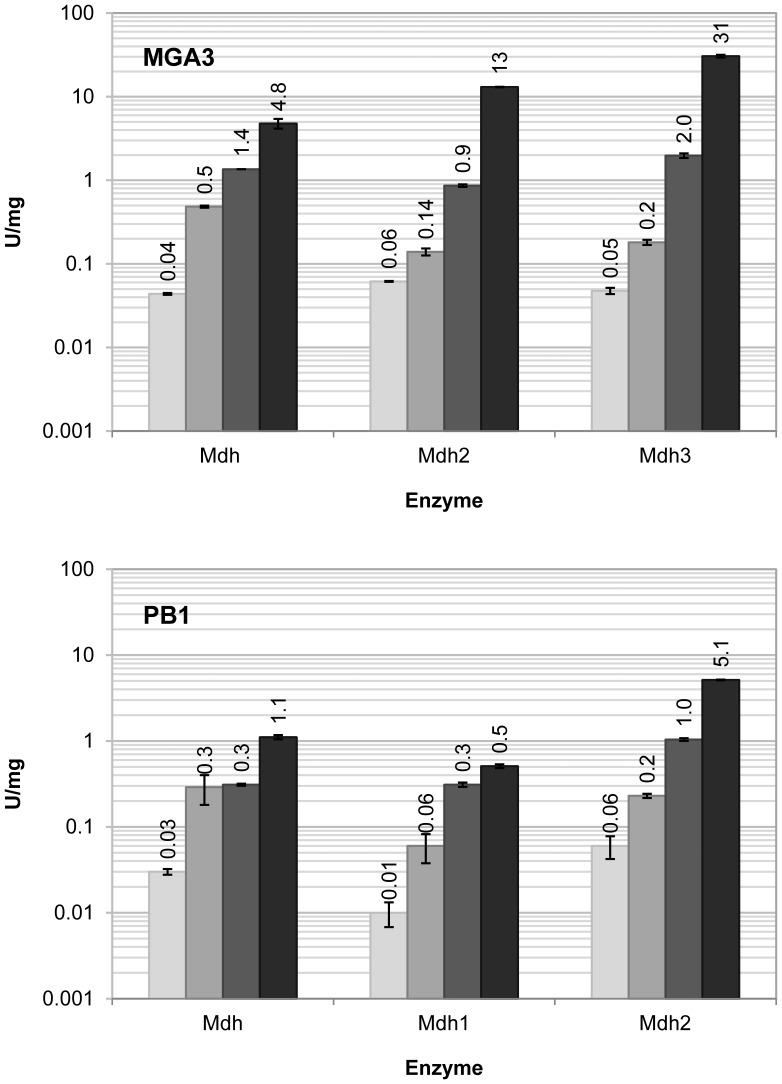
Activation of Mdh by the activator protein ACT. The catalytic activities of Mdh^M^, Mdh2^M^ and Mdh3^M^ from MGA3 (upper panel) and of Mdh^P^, Mdh1^P^ and Mdh2^P^ from PB1 (lower panel) in the absence and presence of ACT. The catalytic activity of the enzymes with methanol (white), methanol+ACT (light grey), ethanol (dark grey), and ethanol+ACT (black) as substrates are shown. The experiments were performed in triplicates with 500 mM alcohols and the mean values with standard errors are indicated.

Both the MGA3 and the PB1 genome sequences had only one *act* gene positioned on the chromosome, similar to the one found in the *B. methanolicus* C1 genome [13], [15]. It was thus of interest to investigate if the respective ACT proteins could stimulate catalytic activity of all Mdh proteins in vitro. To establish reliable conditions, Mdh^M^ was first tested together with Act^M^ at different relative concentrations of the proteins (C_MDH_:C_ACT_ of 20∶1 to 1∶2), using methanol as substrate. Full activation was reached at a relative concentration of between 5∶1 and 1∶1, and no inhibition due to higher activator concentrations was observed (data not shown). For further testing, equal concentrations of Mdh and ACT (1∶1) were used. Next, similar assays were performed with all six Mdhs using methanol as substrate and the data showed that the Mdh activities were increased 2 to 13-fold for the MGA3 Mdhs in the presence of Act^M^, and 3 to 10-fold for the PB1 Mdhs, in the presence of Act^P^ (**Figure 4**).

We then conducted similar analyses using ethanol as the substrate, and the results showed that the catalytic activities were increased 3 to 15-fold for the MGA3 Mdhs and 2 to 5-fold for the PB1 Mdhs (**Figure 4**), in the presence of ACT. When using formaldehyde or acetaldehyde as substrates the presence of ACT caused no significant stimulation of catalytic activities for any of the Mdhs (data not shown). Thus, ACT increases the dehydrogenase versus the reductase activity ratio for all six Mdh proteins in vitro.

### Mdh^M^ can be Catalytically Stimulated by the *B. subtilis* NudF Protein in vitro

The ACT present in both *B. methanolicus* genomes is a member of the nudix hydrolase family [14], [15]. Nudix hydrolase genes are found widespread in bacterial genomes but the *B. methanolicus act* gene is the only member of this family known to encode a protein with the purpose to enhance the activity of other enzymes. The *B. subtilis nudF* gene product, here denoted NudF, displays 61% protein sequence identity to Act^M^, and it has been verified experimentally that NudF belongs to the ADP-ribose pyrophosphatase subfamily [35], [36]. The biological role of NudF in *B. subtilis* remains unclear, but it has been shown to enhance isopentenol synthesis in an *E. coli* strain optimized for isopentenol production [36]. NudF and ACT are identical in motifs defining the Nudix hydrolases group [15]. To broaden our understanding of *B. methanolicus* ACT, we investigated whether NudF could substitute for ACT in activating the *B. methanolicus* Mdhs. To test this, NudF was produced recombinantly and purified. Mdh^M^ was chosen as model protein and its activity tested together with NudF, and the results clearly demonstrated that the Mdh^M^ activity was stimulated equally well (about 8-fold) by NudF as by Act^M^ (data not shown). This implies that the ACT type of proteins could be widespread among the Bacilli and the biological impact of this remained unknown.

### The MDHs have Similar *V_max,MeOH_*, *K_m,MeOH_* and *K_m,NAD_* Values in vitro in Absence of ACT

The purified Mdhs were subjected to in vitro kinetic characterizations to determine *V_max_* and *K_m_* values on selected substrates. The Mdhs are catalytically active on a variety of alcohols (**Figure 2**), but of these *B. methanolicus* MGA3 can only grow on methanol (data not shown). To obtain biologically relevant data, kinetic experiments were therefore conducted by using methanol as substrate. The six Mdh proteins were assayed for initial reaction rates under optimized assay conditions, as described above, and with varying methanol concentrations, and the data showed that they displayed similar kinetics. The *K_m,MeOH_* values were similar and between 170 mM and 360 mM for the MGA3 Mdhs and between 170 mM and 330 mM for the PB1 Mdhs. The corresponding *V_max,MeOH_* values were between 0.06 U/mg and 0.09 U/mg for the MGA3 Mdhs, and between 0.015 and 0.08 U/mg for the PB1 Mdhs (**Table 2**).

**Table 2:** In vitro kinetic constants of Mdhs in the presence and absence of ACT.

	Mdh^M^		Mdh2^M^		Mdh3^M^		Mdh^P^		Mdh1^P^		Mdh2^P^	
	*K_m_*	*V_max_*	*K_m_*	*V_max_*	*K_m_*	*V_max_*	*K_m_*	*V_max_*	*K_m_*	*V_max_*	*K_m_*	*V_max_*
Variable Substrate	(mM)	(U/mg)	(mM)	(U/mg)	(mM)	(U/mg)	(mM)	(U/mg)	(mM)	(U/mg)	(mM)	(U/mg)
Methanol	170±20	0.06±0.002	360±30	0.09±0.003	200±70	0.07±0.005	220±30	0.03±0.001	170±60	0.015±0.001	330±0.05	0.08±0.004
Methanol+ACT	26±7	0.4±0.02	200±20	0.2±0.008	150±10	0.4±0.008	10±1	0.2±0.003	5±1	0.05±0.002	110±50	0.38±0.04
Formaldehyde	1.1±0.2	0.6±0.03	4.5±0.4	1.8±0.06	7.1±0.9	4.6±0.2	3.0±0.2	0.5±0.007	7±1	0.6±0.01	1.0±0.1	1.1±0.03
NAD^+^	∼0.01^a^		0.02±0.005		0.02±0.004							
NAD^+^+ACT	0.02±0.002		0.08±0.02		0.08±0.01							

a The K_m_ value for Mdh^M^ was estimated since the real value is close to the detection limit of the applied method.

At least three independent experiments were performed, and the mean values with standard errors are given.

The *K_m,NAD_* values were only determined for the MGA3 Mdhs, and these were found to be between 0.01 mM and 0.02 mM, ensuring the use of sufficient NAD^+^ when performing the Mdh assay. Together these data indicate that the kinetic constants for all the six Mdhs are relatively similar at the conditions tested.

### The *K_m,MeOH_* Values for Mdh^M^, Mdh^P^ and Mdh1^P^ are Substantially (up to 34-fold) Reduced in the Presence of ACT, While the *K_m,NAD_* Values for the MGA3 Mdhs did Increase (up to 4-fold) under the Same Conditions

The *K_m,MeOH_* was significantly reduced (7-fold) to 26 mM for Mdh^M^ when Act^M^ was added to the reaction, while the corresponding *K_m,MeOH_* value for Mdh2^M^ was reduced slightly (2-fold) and the *K_m,MeOH_* value for Mdh3^M^ remained essentially the same as when tested without Act^M^. For the PB1 enzymes Mdh^P^ and Mdh1^P^ the *K_m,MeOH_* values were substantially reduced (22-fold and 34-fold, respectively) in the presence of Act^P^, while the *K_m,MeOH_* value for Mdh2^P^ was only moderately (3-fold) reduced by Act^P^ (**Table 2**). The Mdh^M^, Mdh^P^ and Mdh1^P^ proteins were listed into one Mdh subtype based on sequence alignments (see above), and the biological impact of these findings is discussed (see below). In experiments using the *B. methanolicus* strain C1, ACT has been shown to have higher influence on the *V_max_* values when Mdh is assayed under physiological methanol concentrations (0.1–1 mM), and the methanol turnover rate was reported to be enhanced up to 40-fold by the addition of ACT [13]. The *K_m,NAD_* values for Mdh^M^, Mdh2^M^ and Mdh3^M^ were also determined in the presence of Act^M^ and observed to be increase 2 to 4-fold compared to when assayed without added ACT (**Table 2**). Although the biological function of this remains to be elucidated, one could speculate if the combination of increased affinity for MeOH and decreased affinity for NAD^+^ in the presence of ACT, might help the cells to keep the NAD^+^/NADH ratio inside the cells stable even at very high methanol concentrations. Based on this, it is plausible to assume that Mdh, together with ACT, may have a particular role for methylotrophy under conditions of low external methanol concentrations.

### The Mdhs Generally have Higher Activity and Affinity for Formaldehyde Compared to Methanol

The biological significance of Mdh for methanol oxidation during methylotrophic growth is unambiguous, while the biological role of these enzymes as part of a formaldehyde detoxification system in the methanol consuming cells is less studied. It was here demonstrated that all enzymes displayed both formaldehyde- and acetaldehyde reductase activities (see above), and we chose to characterize the reductase properties kinetically. By using formaldehyde as the substrate the *K_m,FA_* values were determined to be 1.1 mM, 4.5 mM and 7.1 mM respectively and the corresponding *V_max,FA_* values were 0.6 U/mg, 1.8 U/mg and 4.6 U/mg for the MGA3 proteins Mdh^M^, Mdh2^M^ and Mdh3^M^ respectively (**Table 2**). For the PB1 proteins Mdh^P^, Mdh1^P^, and Mdh2^P^ the K*_m,FA_* values were 3 mM, 7 mM and 1 mM, respectively, and the corresponding *V_max,FA_* values were 0.5 U/mg, 0.6 U/mg and 1.1 U/mg, respectively. Together, these results show that all six Mdhs generally have higher affinity and higher *V_max_* values when formaldehyde is the substrate, compared to when methanol is the substrate. Due to the lack of any response to ACT on the formaldehyde reductase activities of the Mdhs (see above), it was chosen not to perform kinetic measurements in the presence of ACT. The biological relevance of the reduction of formaldehyde by the Mdhs is not known. This could however, be part of additional formaldehyde detoxification system under critically high formaldehyde levels in the cell. From a BLAST search, a protein named EutG, with similarity to the MDHs was found. EutG is a novel Fe-alcohol dehydrogenase, and its main function is suggested to be the protection of cells from aldehydes by converting them into alcohols [37]. Like EutG, all six Mdhs have the ability to catalyze the reduction of formaldehyde and acetaldehyde into its respective alcohols, indicating that they may play a part in detoxification systems.

### The *mdh* Genes are Transcribed at Different Levels in Exponentially Growing *B. methanolicus* Cells

The presence of several alternative MDH-encoding genes in the *B. methanolicus* strains is an interesting attribute. qPCR was used to determine if the Mdhs transcriptional levels were regulated in response to methylotrophic versus non-methylotrophic growth (methanol or d-mannitol). In addition, qPCR was adopted to estimate the relative expression level among the Mdhs located in each of the genomes of the two strains studied. We have previously demonstrated that the *mdh* ^M^ transcription is very high in *B. methanolicus* MGA3 cells, and that it was slightly up-regulated in cells growing on methanol versus on d-mannitol, while the *act* ^M^ transcript levels were similar under both growth conditions [20]. We here included all six Mdhs encoding genes in a similar qPCR analysis, and the results showed that the relative transcription levels of *mdh* ^M^ and *mdh2* ^M^ were 2 and 3-fold higher on methanol compared to on d-mannitol while the *mdh3* ^M^ transcription level was essentially the same under the two different growth conditions. In contrast, analogous experiments using the PB1 strain showed that both *mdh* ^P^ and *mdh1* ^P^ were transcribed at a higher rate (2- and 14-fold, respectively) in cells growing on d-mannitol compared to methanol, while *mdh2* ^P^ was unaffected by the carbon source. The three Mdhs present in each strain could have very different total transcription levels, which again can lead to different amounts of enzymes being expressed. One way to investigate this closer is to compare the difference in the respective Ct values obtained under standardized qPCR conditions for the three genes of each strain (see material and methods). For example, in MGA3 the Ct*_mdh2_* ^M^ minus the Ct*_mdh_* ^M^ was found to be 8 and the Ct*_mdh3_* ^M^ minus the Ct*_mdh_* ^M^ was 14. Taking into consideration that all three primer pairs have about 100% primer efficiency (data not shown), these numbers imply that the *mdh2* ^M^ and *mdh3* ^M^ transcript levels are approximately 60-fold (2^6^) and 4000-fold (2^12^) lower than the *mdh* ^M^ transcript level, respectively. Likewise, the *mdh1* ^P^ and *mdh2* ^P^ transcript levels are approximately 32-fold (2^5^) and 4000-fold (2^12^) lower than the *mdh* ^P^ transcription level, under the conditions tested. The *mdh2* ^M^ and *mdh3* ^M^ coding sequences are 96% identical at the DNA level and to rule out any cross hybridization of the qPCR primers in these experiments, the respective qPCR primer pairs (see Materials and Methods) were tested towards plasmid DNAs, pTMB1 and pTMB2, carrying the *mdh2* ^M^ and *mdh3* ^M^ gene sequences respectively. No detectable PCR products were obtained when the *mdh2* ^M^ specific primers were used together with pTMB2 DNA, or alternatively when the *mdh3* ^M^ specific primers were used together with pTMB1 DNA template (data not shown). These data confirmed that the qPCR primers used for *mdh2* ^M^ and *mdh3* ^M^ are specific for their respective targets, and that the obtained data should be reliable.

Taken together, the individual Mdh-encoding genes are subjected to different transcriptional regulation, as a response to the C-source, and their total transcriptional levels differ from each other. This opens up for a different methanol oxidation potential of the two strains, which may explain why the MGA3 and PB1 strains were found to have highly different CO_2_ profiles in fed batch methanol fermentations [18].

### Conclusions

The presence of paralogous Mdhs was recently discovered when genome sequencing the two *B. methanolicus* strains MGA3 and PB1 [18]. Each of the sequenced strains harbored three Mdh-like genes that fall into two distinct subtypes. Biochemical characterization of all six enzymes revealed that they were able to oxidize a wide range of primary alcohols; however, they showed different biochemical and physical properties and are subjected to distinct regulatory cues. These results imply that methanol oxidation in this methylotrophic bacterium is a complex process involving multiple enzymes, and is presumably regulated at several different levels. Possibly, this complexity could be beneficial for a carefully adjusted response to growth in challenging environments where the temperature and methanol concentrations vary.
